# Referrals, Symptoms and Treatment of Patients Referred to a Secondary Spine Centre—How Can We Help?

**DOI:** 10.3390/jcm12113840

**Published:** 2023-06-04

**Authors:** Ruud Droeghaag, Daphne Nabben, Anouk Smeets, Wouter van Hemert, Narender van Orshoven, Henk van Santbrink, Jasper Most, Inez Curfs

**Affiliations:** 1 Department of Orthopaedic Surgery, Zuyderland Medical Centre, Henri Dunantstraat 5, 6419 PC Heerlen, The Netherlands; d.nabben@student.maastrichtuniversity.nl (D.N.); w.vanhemert@zuyderland.nl (W.v.H.); j.most@zuyderland.nl (J.M.); i.curfs@zuyderland.nl (I.C.); 2 CAPHRI School for Public Health and Primary Care, Maastricht University, P. Debyelaan 25, 6229 HX Maastricht, The Netherlands; h.van.santbrink@mumc.nl; 3 Department of Neurosurgery, Maastricht University Medical Centre, P. Debyelaan 25, 6229 HX Maastricht, The Netherlands; 4 Department of Neurosurgery, Zuyderland Medical Centre, Henri Dunantstraat 5, 6419 PC Heerlen, The Netherlands; 5 Department of Neurology Zuyderland Medical Centre, Henri Dunantstraat 5, 6419 PC Heerlen, The Netherlands

**Keywords:** characteristics, burden of disease, patient-reported outcome measures, secondary spine centre, conservative care, healthcare optimization

## Abstract

Introduction: Spinal disorders are amongst the conditions with the highest burden of disease. To limit the increase of healthcare-related costs in the ageing population, the selection of different types of care for patients with spinal disorders should be optimized. The first step is to investigate the characteristics of these patients and the relationship with treatment. Research Question: The primary aim of this study was to provide insights in the characteristics, symptoms, diagnosis and treatment of patients referred to a specialized spinal health care centre. The secondary aim was to perform an in-depth analysis of resource utilization for a representative subgroup of patients. Methods: This study describes the characteristics of 4855 patients referred to a secondary spine centre. Moreover, an extensive analysis of a representative subgroup of patients (~20%) is performed. Results: The mean age was 58.1, 56% of patients were female, and the mean BMI was 28. In addition, 28% of patients used opioids. Mean self-reported health status was 53.3 (EuroQol 5D Visual Analogue Scale), and pain ranged from 5.8 to 6.7 (Visual Analogue Scale neck/back/arm/leg). Additional imaging was received by 67.7% of patients. Surgical treatment was indicated for 4.9% of patients. The majority (83%) of non-surgically treated patients received out-of-hospital treatment; 25% of patients received no additional imaging or in-hospital treatment. Conclusion: The vast majority of patients received non-surgical treatments. We observed that ~10% of patients did not receive in-hospital imaging or treatment and had acceptable or good questionnaire scores at the time of referral. These findings suggest that there is potential for improvement in efficacy of referral, diagnosis, and treatment. Future studies should aim to develop an evidence base for improved patient selection for clinical pathways. The efficacy of chosen treatments requires investigation of large cohorts.

## 1. Introduction

The majority of people experience at least one episode of spine-related disorder in their lifetime. Spine-related complaints are an enormous global healthcare burden [1]. As an example, back pain is amongst the conditions with the highest burden of disease in terms of years lived with disability (YLD) [2].The prevalence in adults increases to 19–23% by the age of 80 [3]. Since 1980, the global population of people older than 60 years has doubled, and this number is expected to double again by 2050 [4,5,6]. Due to ageing of the population, the number of patients with spinal disorders is increasing exponentially. The increasing incidence of spinal disorders consequently leads to an increase in healthcare-related costs [7,8].

Health care systems must continually cope with fewer resources per patient. Therefore, it is pivotal to continuously evaluate resource utilization in health care pathways in terms of the characteristics of patients, the volume of diagnostics and the specificity of treatments, and ultimately the appropriateness of referrals from primary to specialized care. The first step is to investigate the characteristics of this population and the relationship between these characteristics and indicated treatments.

Currently, patients with spinal disorders are often referred to a secondary spine centre; it is unclear what resources of specialized healthcare, e.g., imaging, specialized treatments, expert opinion, are utilized and required. In many of cases, no anatomical substrate responsible for the patients’ complaints is found, and the majority of patients receive conservative treatment. Some disease-specific demographic research is available, reporting an increase of expenditures for all spine-related inpatient care and an increasing demand for out-patient spinal care [7,9]. One study focussed on specific biopsychosocial characteristics of patients suffering from chronic low back pain and concluded that a multidisciplinary biopsychological approach is needed for this complex category of patients [10]. More comprehensive information about symptoms, diagnostics, and treatment is lacking in these studies.

The aim of this study was to provide a comprehensive understanding of the resources utilized for patients referred to a specialized spinal health care centre. We therefore assessed patient characteristics, reported symptoms, diagnostic methods, diagnoses, and treatments in a one-year cohort of patients with spinal disorders referred to the secondary spine centre of Zuyderland Medical Centre, the Netherlands.

## 2. Materials and Methods

### 2.1. Study Design

A prospective cohort study using the data of all patients that were referred to the secondary spine centre in 2019 was conducted in Zuyderland Medical Centre Heerlen. This study was approved by the local institutional medical ethical committee (Medical Research Ethics Committee Zuyderland, METCZ20210030).

The first aim of the study was to provide a comprehensive characterization of all patients, including their symptoms and diagnosis. The second aim of this study was to perform an in-depth analysis of resource utilization in the specialized spine centre for a representative subgroup of patients. The first 1000 patients visiting the spine centre in 2019 were selected as the subgroup, with additional manual data extraction.

Patient characteristics were assessed by demographics, reported symptoms, and diagnosis. Resource utilization was defined as receiving a specialist’s consultation (all patients), having additional imaging, or receiving specialized treatment.

### 2.2. Study Population and Selection

The study population consists of all adult patients newly referred to the secondary spine centre in 2019. This study was carried out within the Dutch healthcare system, in which the general practitioner functions as a gatekeeper for secondary healthcare; patients cannot consult a medical specialist without a referral from the general practitioner. The only exclusion criterion was documented objection to participate in scientific research.

### 2.3. Patients, Symptoms, and Diagnosis

An independent hospital data specialist conducted a search in the electronic patient records for the year 2019, using reimbursement codes. In the Dutch healthcare system, all patients visiting a hospital receive specific codes for healthcare reimbursement. As all hospital care is reimbursed based on these codes, the coding is double-checked by financial administrators. Identifying patients using these codes minimized the risk of selection bias. Patient demographics, symptoms, and diagnoses are available in the electronic patient records. Symptoms are assessed by questionnaires, which every patient is required to complete before consultation. Diagnosis codes were clustered into diagnosis groups: (1) non-specific spinal complaints (without evident anatomical substrate); (2) complaints as a result of a herniated disc, or radiculopathy in the thoracolumbar region and radiculopathy in the thoracolumbar spine; (3) spinal stenosis in the thoracolumbar region; (4) cervical spinal pathology with neurological complaints; and (5) other diagnoses.

### 2.4. Imaging, Treatment, and Analgesia

Imaging diagnostics, treatment allocation, and analgesic use were manually extracted from the hospital records by RD and DN. Because of the immense workload arising from manual extraction of these data, we decided to collect data for a subgroup of ~20% of patients (*n* = 1008).

A comprehensive overview of the type of collected data can be found in Table 1.

### 2.5. Data Analysis

Data were collected into an anonymised database. *p* values of <0.05 were considered significant, and the analyses were carried out using IBM SPSS statistics 26 [17].

Descriptive statistics (means ± SD, frequencies as %) were performed. To determine whether the subgroup of the in-depth-cohort was representative of the total cohort, we compared their characteristics with the total group. Data were normally distributed and were hence compared by independent sample t-tests and Chi-Square tests for continuous and categorical variables, respectively.

## 3. Results

### 3.1. Patient Characteristics

A total of 4855 patients were referred to the secondary spine centre at Zuyderland Medical Centre the Netherlands in 2019. None had documented objection to participate in research. Patient characteristics are presented in Table 2. Except for age, the subgroup of patients was comparable to the full cohort.

### 3.2. Questionnaire Scores

**Figure 1:**
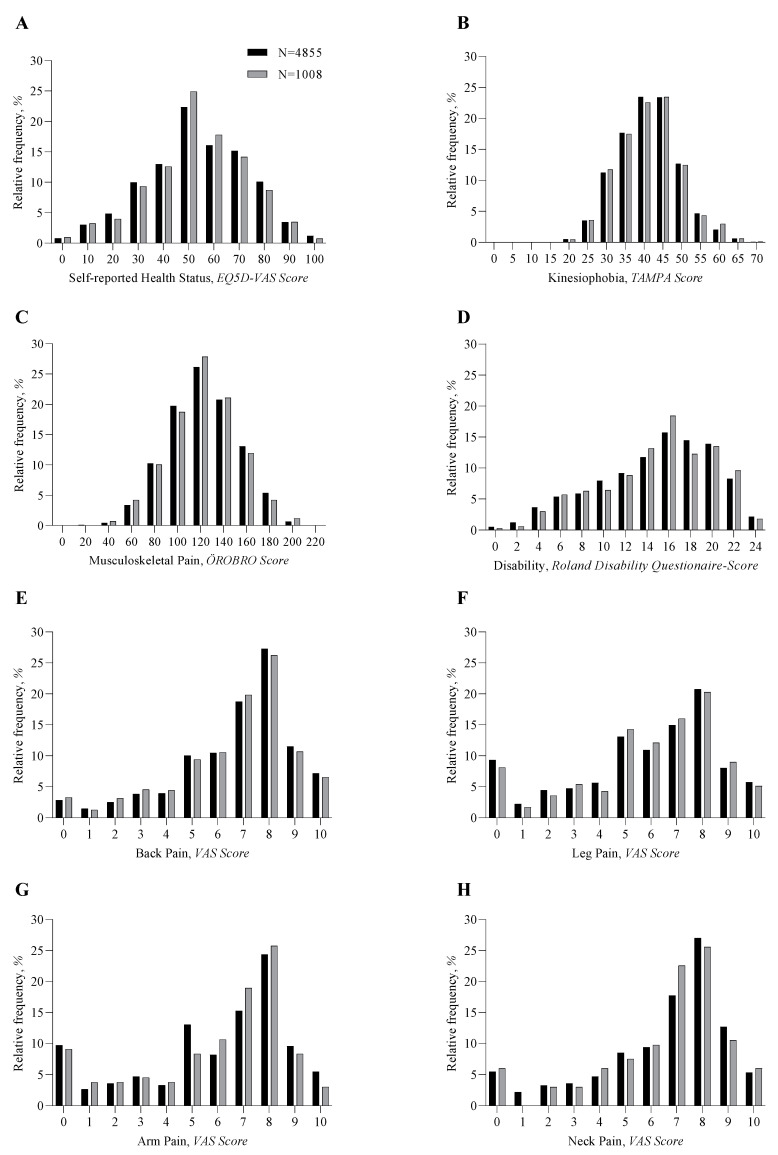
Histograms of questionnaire scores.

Questionnaire scores are summarized in Table 3 and Figure 1. On average, the completion rate of questionnaires was 75% in the total cohort and was comparable to the completion rate in the subgroup. There were no statistically significant differences between the total cohort and the subgroup of patients. Within the total cohort, self-reported health status at first referral was 53.3 ± 20.2 (EuroQol 5D Visual Analogue Scale), musculoskeletal pain was 121.8 ± 30.1 (Örebro Musculoskeletal Pain Screening Questionnaire), disability was 14.3 ± 5.3 (Roland Disability Questionnaire), kinesiophobia was 41.1 ± 8.0 (Tampa Scale of Kinesiophobia), and pain ranged from 5.8 to 6.7 (Visual Analogue Scale neck, back, arm, and leg).

### 3.3. Additional Imaging

All patients referred to the spine centre received conventional radiographic imaging (X-ray) of the spinal region for which they were referred. Of the 1008 patient subgroup, 682 (67.7%) received additional imaging diagnostics. Of these patients, 638 (93.5%) received an MRI scan, and 113 (16.6%) received a CT scan.

### 3.4. Diagnosis

Among the referred subgroup of patients, 315 (31%) were diagnosed with non-specific spinal complaints, 332 (33%) with a herniated nucleus pulposus or radiculopathy in the thoracolumbar region, 110 (11%) with spinal stenosis in the thoracolumbar region, and 75 (7%) with cervical pathology with neurological complaints. A total of 176 (17%) patients received other diagnoses (for example, peripheral mononeuropathy, coxarthrosis, musculoskeletal pathology of the shoulder, etc.).

**Figure 2:**
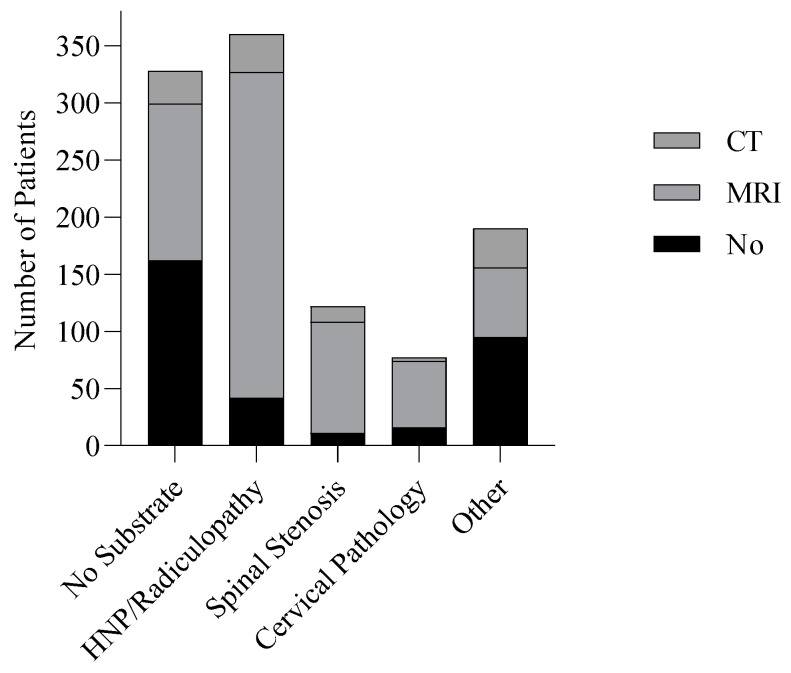
Imaging Diagnostics in Diagnosis Groups.

The use of additional imaging diagnostics varied between diagnosis groups (Figure 2). For the diagnoses ‘Non-specific spinal complaints (No substrate)’ or ‘Other’, additional imaging was utilized in 50% of cases, while for other diagnoses, the utilization of MRI and CT exceeded 90%.

### 3.5. Treatment

**Figure 3:**
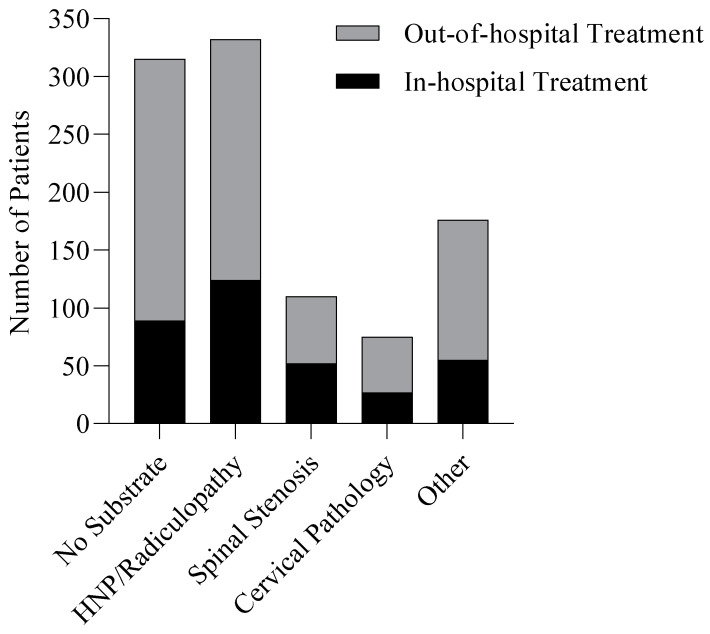
Treatments in diagnosis groups.

Among the referred subgroup of patients, non-surgical treatment was indicated for 959 patients (95%), and 49 patients (5%) were treated surgically (Table 4). Among all diagnosis groups, most patients received out-of-hospital treatment. In-hospital treatments consisted of treatment by a pain specialist (*n* = 200, 20%), rehabilitation (*n* = 67, 7%), referral to another specialist (*n* = 26, 3%), and use of a corset (*n* = 25, 2%). Out-of-hospital treatments consisted of physical therapy (*n* = 441, 44%) and expectant management (i.e., no specific treatment, but only explanation about the condition) or referral back to general practitioner (*n* = 353, 35%). Indicated treatments per diagnosis are provided in Figure 3.

### 3.6. Additional Diagnostics or In-Hospital Treatment

Of all 1008 patients, 238 patients (24%) received a specialist opinion only, without additional diagnostics or in-hospital treatment. Of these patients, the vast majority was diagnosed with non-specific spinal complaints (*n* = 122, 51%), 28 patients (12%) with a herniated nucleus pulposus or radiculopathy in the thoracolumbar region, 7 (3%) with spinal stenosis in the thoracolumbar region, and 11 (5%) with cervical pathology with neurological complaints. Seventy (29%) patients received other diagnoses. Of the 238 patients who did not receive additional diagnostics nor in-hospital treatment, 25–40% (~6–10% of all patients) had acceptable or good questionnaire scores and considerably better scores on leg pain, arm pain, and disability compared to patients that did receive in-hospital treatment or diagnostics. Histograms of the questionnaire scores comparing these groups are available in Supplementary Materials Figure S1.

## 4. Discussion

The objective of this study was to characterize the patient population at a Dutch secondary spine centre. In the Dutch health care system, access to specialized care requires referral by patients’ general practitioners. Our study included all newly referred patients, resulting in a cohort that is representative for daily practice and comparable to other related studies [18,19]. Further we investigated the utilization of resources in the specialized health care centre, namely additional diagnostics (MRI, CT), and specialized treatment, in addition to medical consultation by a specialist.

A substantial number of patients (70%) were overweight (BMI > 25) or obese (BMI > 30), which is 20% higher than the national average. In particular, the proportion of patients classified as obese was larger (30% in our cohort vs. 14% in the total population) [20]. This is representative of the results of a meta-analysis, which showed that overweight and obese patients were more likely to suffer low back pain and had an increased tendency for seeking care [21]. This implies that preventive measures against overweight on the level of the population could have a direct impact on spinal care. Although are results on BMI are in line with the findings of previous studies, it could be possible that our results are biased due to missing data. For all patients that received any type of surgical treatment in our hospital, BMI was available. For all other patients, BMI was only available sporadically. If the patients for which data was available had a higher BMI than the patients for which no data was available, we might have overestimated the overweight and obesity in the total sample.

The disease burden in our population is supported by analgesic use and the reported symptoms of the referred population. In our study, we found that ~80% of patients reported using analgesics, of which around one-third used opioids, at the time of referral from primary care. These findings are in line with the findings of a disease-specific study performed by Ashworth et al., in which opioid prescription for low back pain in primary care was found to be 30% [22].

The impact of disease is also evident when comparing questionnaire scores about disability and quality of life to the healthy population or other serious diseases. For example, the mean self-reported health status, as assessed by EQ-5D VAS score on a scale to 100, was 53.3 among our study population, as compared to ~75 for an age-matched general population [11]. Scores were slightly worse in our population than scores of other study populations, for example, patients suffering from chronic low back pain [23]. The mean VAS back pain score was 6.7 and was even higher in patients suffering from non-specific spinal complaints (7.3) in our cohort.

The high analgesics use and severe symptoms observed in our cohort strongly indicate the need for specialist care due to the high burden of disease for the population. The vast majority of patients referred to the secondary spine centre received non-surgical treatment, most often carried out outside of hospital; e.g., physical therapy, referral back to GP, or expectant management. In addition, a quarter of all newly referred patients did not require specific in-hospital diagnostics or treatment. Based on our data, it remains unclear for which proportion of these patients a referral to secondary care might have been unnecessary or inappropriate.

To enhance the efficacy of the pathway to diagnosis and treatment, it would be advantageous to be able to profile patients based on characteristics at the time of referral. However, the generally small differences and variability in characteristics and reported symptoms (by questionnaires) impede such statistical patient profiling. Although several studies on non-surgical treatment are available, not many selection criteria for patient profiling have been identified [1]. For example, the Lancet series on low back pain series showed that most distinguishing criteria have limited diagnostic accuracy [24]. Well-powered clinical research on the effects of non-surgical treatments for the studied population is deficient to enable detailed patient profiling. Insight in the effectiveness of such treatments for different diagnoses and subgroups of patients could drive forward our understanding of this complex category of patients and ameliorate patient selection for different types of treatments in primary and secondary healthcare.

Other studies have initiated revision of classic patient pathways and have generated promising results. One study investigated the efficacy of ‘Primary Care Plus’ for spine-related complaints. In this study, patients that would normally be referred to secondary spinal care received multidisciplinary out-of-hospital consultation with standardised anamnesis, physical examination, and diagnostics focussed on red flags. Patients with suspected severe pathology were then referred to secondary care. Of all patients consulting Primary Care Plus, only ten percent required referral to secondary care. This is beneficial to patients, healthcare providers, and society in general, as it leads to a significant reduction of time to diagnosis, while also reducing healthcare related costs [25]. A previously published study form Wilgenbusch et al. found that a co-ordinated pathway for referral of patients with low back pain resulted in over 50% more surgery candidates than the conventional referral process [26]. In our cohort, only 4.9% of patients were treated surgically. With the implementation of more strict pathways for referrals, the proportion of patients receiving in-hospital diagnostics and treatment might increase significantly. For these patients with severe symptoms with an identified anatomical substrate, as indicated by the necessity to treat surgically, questionnaire scores were indeed significantly worse than patients who were treated non-surgically.

In addition to focusing on the optimization of the pathway to surgical intervention, it is important to provide the best possible care for the large number of patients that do not require surgical intervention but are insufficiently aided by conservative treatment centred around physical complaints (i.e., physical therapy). Especially for this group of patients, a systematic approach incorporating not only physical but also psychosocial factors and integrated workplace strategies is of paramount importance. Although more data are needed, it seems that the development and implementation of specific healthcare pathways integrating these factors could increase the effectiveness and cost-effectiveness of conservative care [27].

### Strenghts and Limitations

The main strength of this study is the size of the investigated cohort and the level of detail of the data used. All data were collected at the moment of the first outpatient visit at the spine centre. This type of data collection and analysis provides representative insight into the actual day-to-day healthcare, as opposed to prospective trials and randomised controlled trials.

This prospective cohort study is limited by several constraints. The data used in this study were collected at the time of the first outpatient visit after referral. The missing data and the use of patient reported questionnaires could potentially lead to a selection bias [28]. Moreover, referral patterns and treatment strategies are region- and healthcare-system-specific, which may impact the generalizability of the study.

While our cohort consisted of a large sample size of nearly 5000 patients, the data regarding treatments, analgesics use, and imaging diagnostics were manually extracted from the hospital records and were limited to the first 1008 patients of this cohort. We limited this detailed investigation due to the workload associated with this manual extraction, and thus these data do not necessarily reflect the outcomes of the entire cohort. However, based on demographics and questionnaires, the smaller cohort was comparable to the full cohort and, therefore, likely representative. For future studies on more advanced patient profiling, even larger sample sizes are required, because subgroups with less frequent indications are too small for considering covariates, e.g., demographic variables.

## 5. Conclusions

This prospective cohort study provides insight in the characteristics of patients with spinal disorders referred to a secondary spine centre. The burden of disease among these patients was high, and a large group of patients used opioids to relieve their complaints. Only a select group of patients was treated surgically, whereas over 90% of patients were treated non-surgically. One-third of patients did not receive additional imaging diagnostics. The vast majority of conservatively treated patients received out-of-hospital treatment. Although we found several statistically significant differences in characteristics between groups of patients receiving different treatments, we found no variables that were sufficiently specific to aid in patient profiling. Even though the outcomes of our study suggest that there is relevant potential for improvement of efficacy of referral, diagnosis, and treatment, for example, by triaging referrals, educating referring doctors, and organising multidisciplinary out-of-hospital consultation, our lack of knowledge on the effectiveness of care pathways for different categories of patients impedes further healthcare optimization.

Large prospective observational cohort studies or randomized controlled trials investigating the relationship between patient characteristics and effectiveness of new healthcare pathways, including non-surgical treatments, are mandatory for further developing healthcare allocation and conservative care for patients suffering from spinal complaints.

## Figures and Tables

**Table 1:** Overview of extracted data.

Data	Measure	Variable
Patient characteristics	Age	Years
	Gender	M/F
	BMI	Kg/m^2^
	Smoking	Yes/No
	Duration of symptoms	Weeks
	Analgesic use	Yes/No (If yes → paracetamol/NSAID/opioids/ neuropathic pain medication)
Questionnaires	EQ-5D VAS	Score: 0–100 (High score equals better health status) Acceptable: >70; Good: >80 [11]
	RDQ	Score: 0–24 (High score equals more disability) Acceptable: <6; Good <4 [12]
	Tampa Scale of Kinesiophobia	Score: 17–68 (High score equals more kinesiophobia) No kinesiophobia: <37 [13]
	VAS back/neck/leg/arm	Score: 0–10 (High score equals more pain) Acceptable: <5; Good: <1 [14]
	ÖREBRO	Score 0–210 (High score equals more pain) Acceptable: <130; Good: <105 [15,16]
Additional diagnostics	Additional imaging	MRI/CT
	Other additional diagnostics	EMG/Diagnostic nerve block
Diagnosis codes	Diagnosis groups	Non-specific spinal complaints
		Complaints as a result of a herniated disc, or radiculopathy in the thoracolumbar region and radiculopathy in the thoracolumbar spine
		Spinal stenosis in the thoracolumbar region
		Cervical spinal pathology with neurological complaints
		Other diagnoses
Treatment data	Intervention	Surgery (and type of surgery, e.g., interbody fusion, interlaminar decompression, discectomy, laminectomy, foraminotomy, sacroiliac joint fusion)/Conservative (and type of conservative treatment, e.g., physical therapy, pain treatment, expectative, rehabilitation, return to general practitioner)

Abbreviations: BMI: Body Mass Index, EQ-5D VAS: EuroQol 5D Visual Analogue Scale, MD: Missing Data, ÖREBRO: Örebro Musculoskeletal Pain Screening Questionnaire, RDQ: Roland Disability Questionnaire, TAMPA: Tampa Scale of Kinesiophobia, VAS: Visual Analogue Scale.

**Table 2:** Patient Characteristics.

Name	Factor	Outcome (*n* = 4855)	%MD	Outcome (*n* = 1008)	%MD
Personal and demographic	Age	58.1 ± 15.4	0%	60.0 ± 14.1	0%
	Gender (male/female)	2146/2709 (44/56%)	0%	445/563 (44/56%)	0%
	Body Mass Index (kg/m^2^) Overweight, Obesity	28.1 ± 5.3	76%	28.2 ± 5.4	78%
		41%, 31%		36%, 33%	
	Smoking	-	-	34.1% Yes	59%
				65.9% No	
	Duration of symptoms	-	-	<6 w 12.5%	6%
				6 w–3 m 17.9%	
				3 m–6 m 14.6%	
				6 m–12 m 14.0%	
				>12 m 35.0%	
Analgesics use	Opioids *	-	-	27.9%	0%
	NSAIDs **	-	-	25.7%	0%
	Paracetamol ***	-	-	29.7%	0%
	Co-analgesics	-	-	6.9%	0%
	None	-	-	22.8%	0%
	Not reported	-	-	18.3%	0%

Abbreviations—MD: Missing Data, NSAID: Non-Steroidal Anti-Inflammatory Drug. * With or without NSAIDs/paracetamol/co-analgesics. ** Without opioids; with or without paracetamol/co-analgesics. *** Without NSAIDs or opioids; with or without co-analgesics.

**Table 3:** Questionnaire scores.

Factor	Outcome (*n* = 4855)	%MD	Outcome (*n* = 1008)	%MD
EQ-5D VAS (0–100)	53.3 ± 20.2	15%	53.1 ± 19.5	13%
RDQ (0–24)	14.3 ± 5.3	37%	14.4 ± 5.1	31%
TAMPA (20–68)	41.1 ± 8.0	24%	41.1 ± 8.2	17%
VAS Back (0–10)	6.7 ± 2.3	25% *	6.6 ± 2.4	22% *
VAS Leg (0–10)	5.8 ± 2.8	25% *	5.9 ± 2.7	22% *
VAS Neck (0–10)	6.4 ± 2.6	25% **	6.5 ± 2.6	19% **
VAS Arm (0–10)	5.9 ± 2.8	25% **	5.9 ± 2.8	19% **
ÖREBRO	122 ± 30	57%	121 ± 30	56%

Data are presented as means +/− SD. Abbreviations—EQ-5D VAS: EuroQol 5D Visual Analogue Scale, MD: Missing Data, ÖREBRO: Örebro Musculoskeletal Pain Screening Questionnaire, RDQ: Roland Disability Questionnaire, TAMPA: Tampa Scale of Kinesiophobia, VAS: Visual Analogue Scale. There were no statistically significant differences between groups. * The percentage reflects the missing data for patients referred with complaints of the thoracolumbar spine. ** The percentage reflects the missing data for patients referred with complaints of the cervical spine. The percentages of missing data for all other questionnaires reflect the total cohorts of patients (4855 and 1008).

**Table 4:** Indicated treatment between diagnosis groups.

	Non-Specific Spinal Complaints (*n* = 315)	HNP and Radiculopathy Thoracolumbar Spine (*n* = 332)	Thoracic or Lumbar Spinal Stenosis (*n* = 110)	Cervical Pathology with Neurological Complaints (*n* = 75)	Other Diagnoses (*n* = 176)
Surgery	2 (0.6%)	17 (5%)	14 (13%)	2 (3%)	14 (8%)
Physical therapy	166 (53%)	144 (43%)	32 (29%)	31 (41%)	68 (39%)
Pain specialist	33 (10%)	97 (29%)	31 (28%)	19 (25%)	20 (11%)
Rehabilitation	33 (10%)	14 (4%)	5 (5%)	2 (3%)	13 (7%)
Expectant management	40 (13%)	77 (23%)	12 (11%)	28 (37%)	26 (15%)
General practitioner	82 (26%)	32 (10%)	21 (19%)	3 (4%)	32 (18%)
Corset	13 (4%)	0 (0%)	5 (5%)	0 (0%)	7 (4%)
Referred to another specialist	9 (3%)	3 (1%)	4 (4%)	2 (3%)	8 (5%)
Other treatment	7 (2%)	13 (4%)	7 (6%)	4 (5%)	11 (6%)

## Data Availability

Not applicable.

## Supplementary Materials

The following supporting information can be downloaded at: https://www.mdpi.com/article/10.3390/jcm12113840/s1, Figure S1, Histograms of questionnaire scores, comparing patients receiving in-hospital care and no in-hospital care.

## Abbreviations

BMI	Body Mass Index
CT	Computed Tomography
EQ-5D VAS	EuroQol 5D Visual Analogue Scale
GP	General Practitioner
HNP	Herniated Nucleus Pulposus
MD	Missing Data
MRI	Magnetic Resonance Imaging
NSAID	Non-Steroidal Anti-Inflammatory Drug
ÖREBRO	Örebro Musculoskeletal Pain Screening Questionnaire
QoL	Quality of Life
RDQ	Roland Disability Questionnaire
TAMPA	Tampa Scale of Kinesiophobia
VAS	Visual Analogue Scale
YLD	Years Lived with Disability
