# Supplementary material for: Referrals, Symptoms and Treatment of Patients Referred to a Secondary Spine Centre—How Can We Help?
Source: J Clin Med. 2023 Jun 4;12(11):3840. doi: 10.3390/jcm12113840 (PMC10253721; doi:10.3390/jcm12113840)

Figure S1. Histograms of questionnaire scores, comparing patients receiving in-hospital care and no in-hospital care.

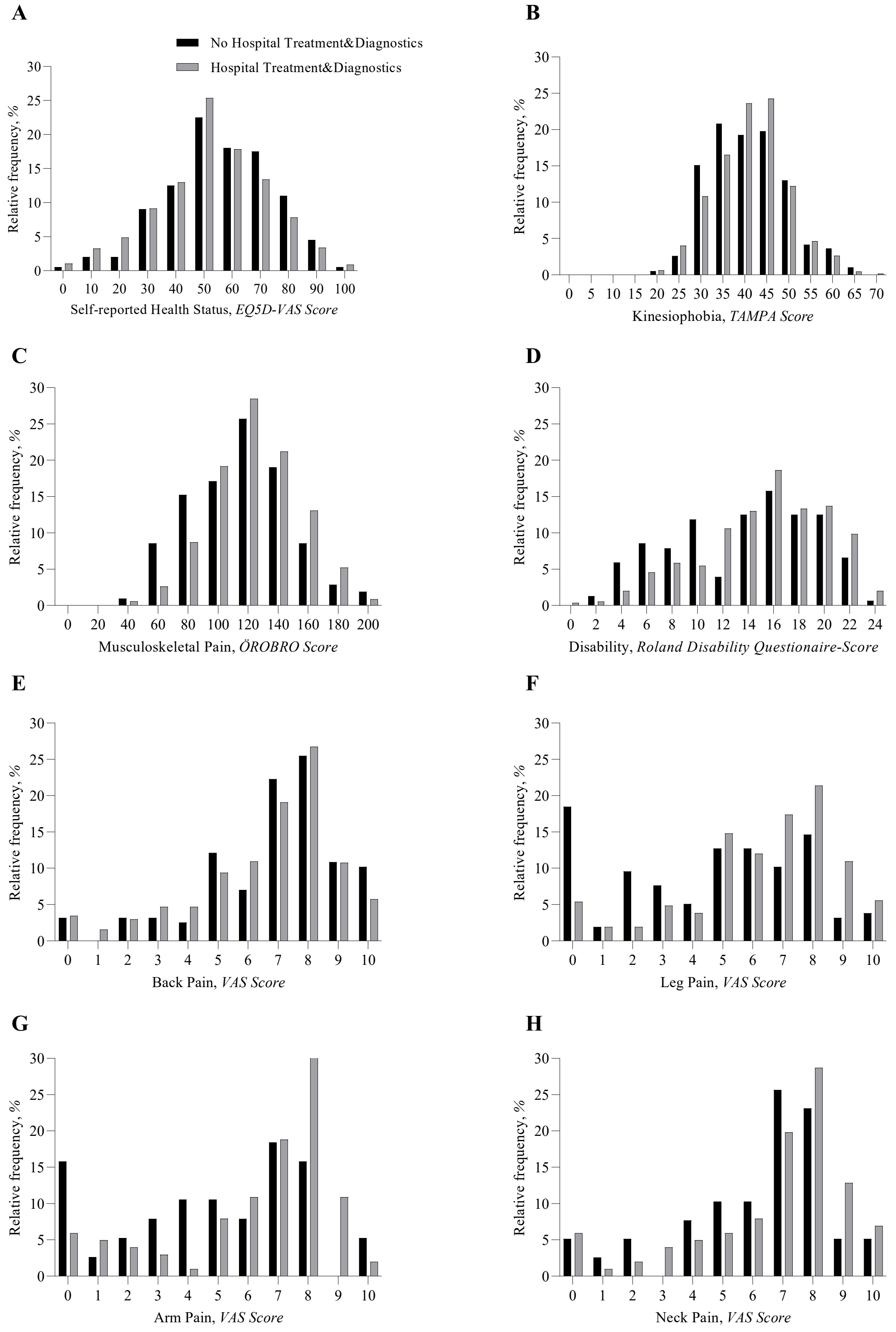

Supplement: Supplementary file 1 [file jcm-12-03840-s001.zip › jcm-2388515-supplementary.pdf]
